# Utilizing Inertial Measurement Units for Detecting Dynamic Stability Variations in a Multi-Condition Gait Experiment

**DOI:** 10.3390/s24217044

**Published:** 2024-10-31

**Authors:** Yasuhirio Akiyama, Kyogo Kazumura, Shogo Okamoto, Yoji Yamada

**Affiliations:** 1 Faculty of Textile Science and Technology, Shinshu University, Nagano 386-8567, Japan; 2 Department of Mechanical Systems Engineering, Nagoya University, Aichi 464-8603, Japan; 3 Department of Computer Science, Tokyo Metropolitan University, Tokyo 191-0065, Japan; 4 National Institute of Technology, Toyota College, Toyota 471-0067, Japan

**Keywords:** gait stability, margin of stability, Lyapunov exponent, inertial measurement unit, convolutional neural network

## Abstract

This study proposes a wearable gait assessment method using inertial measurement units (IMUs) to evaluate gait ability in daily environments. By focusing on the estimation of the margin of stability (MoS), a key kinematic stability parameter, a method using a convolutional neural network, was developed to estimate the MoS from IMU acceleration time-series data. The relationship between MoS and other stability indices, such as the Lyapunov exponent and the multi-site time-series (MSTS) index, using data from five IMU sensors placed on various body parts was also examined. To simulate diverse gait conditions, treadmill speed was varied, and a knee–ankle–foot orthosis was used to restrict left knee extension, inducing gait asymmetry. The model achieved over 90% accuracy in classifying MoS in both forward and lateral directions using three-axis acceleration data from the IMUs. However, the correlation between MoS and the Lyapunov exponent or MSTS index was weak, suggesting that these indices may capture different aspects of gait stability.

## 1. Introduction

The ability to walk deteriorates with age or disease, increasing the risk of falls, particularly among the elderly [1]. Falls in this population can lead to severe injuries, such as fractures, or even prolonged immobility [2]. Although various fall prevention strategies have been proposed, a definitive solution remains elusive. Traditional methods for assessing gait and fall risk, such as the Timed Up and Go Test [3], generally rely on task-based assessments, limiting their applicability in daily living environments. Furthermore, many studies have identified physical parameters influencing fall risk by analyzing recovery motion in response to gait perturbations [4,5,6]. However, parameters such as recovery step length and step time are challenging to measure outside of safety environments.

Recent advancements in wearable technology, particularly small inertial measurement units (IMUs), have enabled the measurement of physical activity in daily environments [7]. IMUs, which can be worn over clothing and record data during movement, are well suited for gait analysis in daily living environments. Many gait evaluation methods using IMUs have been developed, focusing on estimating various gait parameters from the time-series data of body acceleration and angular velocity. These parameters include gait event timing, step length, and step time [8]. Recent studies have also explored the estimation of musculoskeletal parameters, including joint angles, moments, and ground reaction forces [9,10,11]. In addition to traditional signal processing techniques, machine learning methods such as support vector machines and deep learning have been widely employed for parameter estimation [12]. However, the optimal signal components of IMUs remain an open question.

The margin of stability (MoS) is an indicator developed to assess kinematic balance during walking [13]. It quantifies the margin to a fall by calculating the position and velocity vectors of center of mass (CoM) within the support polygon. Originally, MoS estimation required large-scale motion capture systems, limiting its application in daily living environments. However, with the integration of IMUs and machine learning, it is now possible to estimate MoS from gait parameters, enabling assessments in more practical settings.

Several methods exist for estimating the MoS using IMUs. One widely adopted approach involves estimating the CoM and MoS of the human body based on biomechanical analysis. By integrating time-series acceleration signals from various body segments, particularly the pelvis, body segment motion can be estimated kinematically [14,15]. Additionally, by combining IMUs with a musculoskeletal model of the human body, it is possible to estimate the posture of the entire body [16]. Although these methods can estimate the CoM and MoS, the need for motion calibration or the precise placement of IMUs on the body diminishes the overall convenience of using IMUs. An alternative approach involves the use of statistical methods. For example, regression analysis has been employed to estimate the MoS from acceleration signals captured from various body parts [17,18]. Although the second derivative of marker positions was used in place of actual acceleration data, the statistical approach has demonstrated the potential to estimate MoS effectively.

Another widely used index for evaluating dynamic gait stability is the maximum Lyapunov exponent ($\lambda_{s}$) [19]. This metric quantifies stability by constructing a limit cycle from the time-series data of gait motion and measuring the divergence of trajectories in the state space. Several studies have investigated the relationship between gait parameters and the Lyapunov exponent. In many cases, an increase in gait speed is associated with an increase in $\lambda_{s}$, calculated using joint angle patterns [20] as well as marker positions, velocities, and accelerometer data [21]. However, the effects of stride length and stride frequency on $\lambda_{s}$, calculated from markers on the pelvis, remain unclear [22]. Furthermore, by analyzing acceleration data from multiple body segments, it is possible to capture both individual body movements and their coordination during gait. Due to the variety of parameters that can be used to calculate $\lambda_{s}$, it remains unclear which parameter is most suitable for constructing $\lambda_{s}$ in the analysis of gait motion.

The aim of this study is to develop indicators of kinematic stability from IMU-derived acceleration time-series data and to detect changes in gait. While the MoS provides a quantitative measure of kinematic stability, its calculation typically requires whole-body posture data, which are challenging to obtain in daily environments. This study proposes a method to estimate the MoS using a convolutional neural network (CNN) based on IMU acceleration data, making MoS estimation feasible in real-world settings. Additionally, we explore the use of the Lyapunov exponent to detect changes in gait periodicity, further contributing to the development of a practical, wearable gait evaluation system for daily use. The proposed method has the potential to reduce the number of IMUs required compared with the inverse kinematic method and enables the detection of gait pattern changes in real time.

## 2. Methods

### 2.1. Apparatus

A system was developed to induce gait perturbations during steady walking by implementing speed changes on a treadmill (OHTAKE-ROOT Co., Ltd., Ichinoseki, Japan) that adjusts speed in response to an analog voltage controller. During the trials, the treadmill alternated between accelerating by 2 km/h from a steady pace and decelerating back to the baseline speed at 30 s intervals. To ensure safety, participants wore a harness around their torso.

To simulate impaired gait, a knee–ankle–foot orthosis (KAFO) (Gait Innovation, Pacific Supply Co., Ltd., Daito, Japan) was attached to the left leg. This device allowed for adjustable restrictions on the range of motion of the knee and ankle joints. For this study, the maximum knee extension angle was limited to 145 degrees to simulate gait impairment similar to that caused by osteoarthritis.

An optical motion capture system with eight cameras (OptiTrack, NaturalPoint, Inc., Corvallis, OR, USA) was used to record full-body movements. Reflective markers were placed at 24 locations across the body (i.e., front head, rear head, top head, shoulders, elbows, wrists, sternum, anterior superior iliac spines, posterior superior iliac spines, greater trochanters, knees, ankles, toes, and heels). Additional markers were placed on the inner elbows, wrists, knees, and ankles for musculoskeletal model scaling, and a T-pose measurement was taken prior to the walking trials. Cluster markers were used on the left leg during trials with the KAFO to account for the concealment of ankle and knee markers. Heel strike and toe contact events were detected using pressure sensors (FSR-406, Interlink Electronics Inc., Yokohama, Japan) embedded in the insoles.

To calculate gait metrics, IMUs (TSND 151, ATR-Promotions Inc., Kyoto, Japan) were attached to six body locations: the back, pelvis, both thighs, and both feet. These IMUs recorded three-axis acceleration data. The IMUs at the back and pelvis were used to calculate the Lyapunov exponent from acceleration data, while data from the thighs and feet were included to capture coordinated body movements. The IMUs were aligned with the body’s anterior–posterior, lateral, and vertical axes, but no calibration or compensation was performed to correct for misalignment due to individual body shapes considering the applicability in daily living environment.

### 2.2. Participants

The study included nine healthy male participants (age: 23.2 ± 1.5 years, height: 170.3 ± 5.6 cm, weight: 62.3 ± 5.1 kg), all of whom provided written consent.

### 2.3. Protocol

After providing consent, participants changed into suitable clothing and wore a safety harness. The KAFO was adjusted to fit each participant. A comfortable walking speed was established for each participant by gradually increasing the treadmill speed in 0.1 km/h increments, both with and without the KAFO. Participants practiced walking on the treadmill for approximately 5 min under both conditions to become accustomed to treadmill walking, followed by a 10 min rest before data collection. Then, the IMU sensors and motion capture markers were attached to the participants’ bodies.

In this experiment, three different trials were conducted. The acceleration and deceleration (AD) trial involved walking without the KAFO, with repeated accelerations and decelerations, starting from a comfortable walking speed and increasing by 2 km/h. The first acceleration occurred 40 s after the start of walking, followed by five cycles of acceleration and deceleration, each lasting 30 s. The acceleration and deceleration with device (ADD) trial involved walking with the KAFO on the left leg, following the same acceleration and deceleration pattern as the AD trial, where the speed was increased by 2 km/h from the comfortable baseline speed while wearing the KAFO. Finally, the steady walking with device (SD) trial involved steady walking with the orthosis on the left leg at the comfortable speed determined in the no-orthosis condition, where participants maintained a constant speed for 130 s. The time duration of each trial was set to obtain the sufficient number of steps.

The first 10 s of data were excluded from analysis to account for the initial walking phase. In the AD and ADD trials, 330 s of data were analyzed, and 120 s were analyzed in the SD trial. As both the AD and ADD trials included normal and fast speeds, 150 and 180 s of gait data were analyzed for each speed. Additionally, the 10 strides immediately following each speed change were separated as the $v_{c}$ to $v_{a}$ and $v_{a}$ to $v_{c}$ conditions for the analysis of gait indices, as described below. Each of these conditions comprised approximately 50 s of gait motion. The order of the trials was randomized, but the two KAFO trials were conducted consecutively.

### 2.4. Data Processing

Motion capture data were collected at 100 Hz and smoothed using a Butterworth filter with a 6 Hz cutoff frequency. Joint angles and CoM positions were calculated using the musculoskeletal simulator SIMM (MusculoGraphics Inc., Mountain View, CA, USA). IMU data were sampled at 200 Hz and filtered with a 5 Hz Butterworth filter. Ground contact timing for stride extraction and gait cycle calculation was obtained using footswitches.

The MoS in both the forward and lateral directions was calculated at each toe contact (TC) event. The front edge of the base of support was defined as the toe marker’s anterior coordinate, while the lateral edge was defined by the ankle marker.

Gait analysis was conducted across five conditions, corresponding to different speeds and attachments. Gait parameters were defined as follows in this study: Step length was the distance between the ankle markers in the anterior–posterior direction during either the left or right step. Step width was defined as the distance between the ankle markers in the mediolateral direction at heel contact. Stride time was defined as the time interval between consecutive heel contacts (HCs) of the same leg. Step time was defined as the time interval between the heel contact of the left leg and the heel contact of the right leg.

### 2.5. Development of Gait Indices

#### 2.5.1. MSTS Index

To calculate the Lyapunov exponent, acceleration data from a single body location (typically the pelvis or spine) are typically used [23,24]. In this study, this approach was extended by using data from multiple IMUs to define the multi-site time-series (MSTS) index. The MSTS index was calculated similarly to the Lyapunov exponent but used data from all five IMUs. In this study, the embedding dimension used to calculate the MSTS index and the Lyapunov exponent was uniformly set to 5 [25].

Two MSTS patterns were analyzed: Pattern A (back) and Pattern B (pelvis), with corresponding acceleration data from the lower limbs. Thus, five IMUs were used in each pattern. Additionally, the Lyapunov exponent was calculated using pelvic acceleration data.

When calculating the Lyapunov exponent and MSTS index for any given HC event, acceleration data from the subsequent 10 strides were used. The axis corresponding to the direction of the acceleration data was used to calculate indices for both the anterior–posterior and mediolateral directions. This calculation was performed for each HC event, generating stride-by-stride MSTS indices.

To explore the relationship between these indices and the MoS, a correlation analysis was conducted between the MSTS index calculated from a given step and the MoS values for all steps within the 10 strides following that HC event. The MSTS index was calculated for nine conditions, including the SD condition and four AD and ADD conditions, which encompassed the comfortable walking speed ($v_{c}$) and accelerated speed ($v_{a}$) phases, as well as the transitions between these speeds (i.e., $v_{c}$ to $v_{a}$ and $v_{a}$ to $v_{c}$). The Wilcoxon signed-rank test was then performed to compare seven combinations, such as AD ($v_{c}$) vs. AD ($v_{a}$), AD ($v_{c}$ to $v_{a}$), AD ($v_{a}$ to $v_{c}$), and SD; and ADD ($v_{c}$) vs. ADD ($v_{a}$), ADD ($v_{c}$ to $v_{a}$), and ADD ($v_{a}$ to $v_{c}$). The significance level was adjusted using the Bonferroni correction, and the same comparisons were applied to the Lyapunov exponent.

#### 2.5.2. Binary Classification Using CNN

The three-axis acceleration time-series data recorded by IMUs were normalized to 101 frames, corresponding to 0–100% of each stride. The data were then normalized to 8-bit values and converted into RGB images, where each color channel represented anterior–posterior, mediolateral, and vertical acceleration, respectively. The image resolution was determined by the number of sensors and the 101 frames, and these images were used as an input for the CNN.

In this study, the CNN method was employed to perform binary classification, determining whether the MoS at TC was above or below a specified threshold. The network architecture started with a convolutional layer that applied 16 filters of size 3 × 3, followed by a 2 × 2 max pooling layer. Two additional convolutional layers with 32 and 64 filters, respectively, were then applied, each followed by a ReLU activation function and another max pooling layer. A dropout layer with a rate of 0.5 was added after the pooling and fully connected layers. The network was flattened before a fully connected layer with 128 units was applied, followed by another ReLU activation. Finally, the model concluded with a dense layer containing one unit, using a sigmoid activation function for binary classification. The binary cross-entropy loss function was used, and the ADAM optimizer [26] was selected for training. To mitigate potential variability due to data imbalance, model performance was evaluated using 5-fold cross-validation. Data from all participants and conditions were used during model training, with the number of training epochs being set to 50.

Two distinct boundary values were set for both the forward and lateral directions, and the MoS was classified into high and low categories. In this study, the low category was considered positive, while the high category was treated as negative. The thresholds for the MoS were set at 0 m and −0.1 m in the forward direction and 0.1 m and 0.13 m in the lateral direction to assess the sensitivity of the estimation model to different threshold values.

## 3. Results

### 3.1. Gait Parameters

Table 1 shows the walking speeds of the participants under each condition. According to the experimental design, the speed for the SD trial was the same as the AD ($v_{c}$), and the $v_{a}$ condition was 2 km/h faster than the corresponding $v_{c}$ condition. A consistent trend observed across all participants was that the comfortable walking speed in the non-orthosis condition was higher compared with the orthosis conditions.

**Figure 1:**
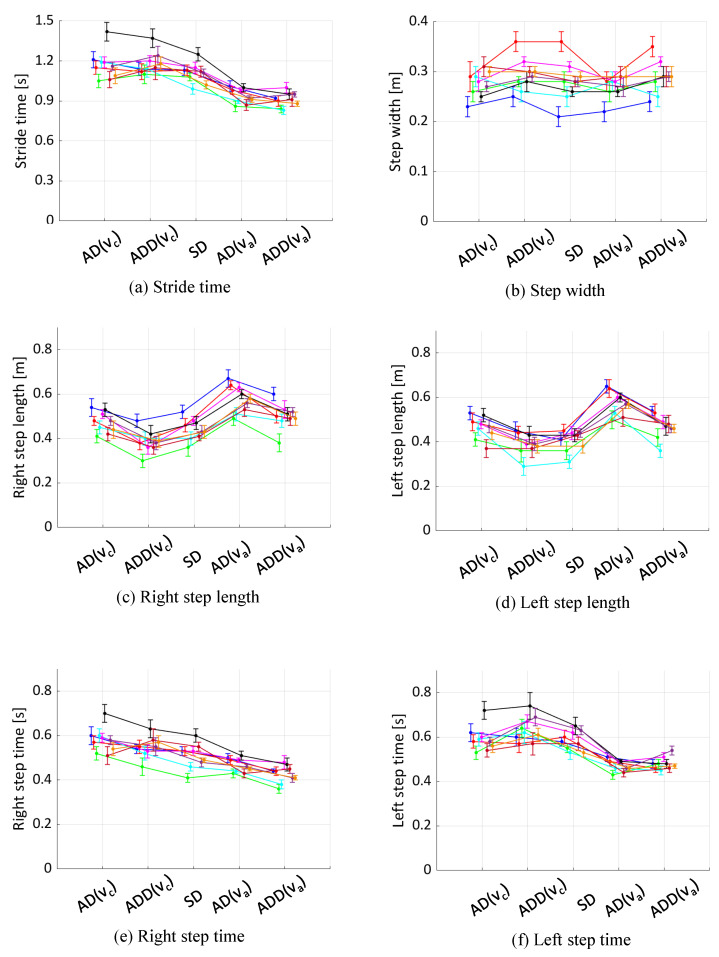
Comparison of gait parameters for participants across conditions. Each plot represents data from an individual subject. The x-axis positions of each sample are offset slightly for improved readability. The plotted values represent the mean, with error bars indicating the standard deviation.

The parameters related to the steps and strides of each participant under different conditions are shown in Figure 1. A common trend observed across all participants is that as walking speed increased, stride time decreased, and step length increased on both the left and right sides. In the KAFO-wearing conditions, step length was reduced for both sides compared with the non-orthosis condition, although this was not consistently reflected in step time. Additionally, the differences in step length and step time between the left and right sides were not pronounced, even in the orthosis-wearing condition.

**Figure 2:**
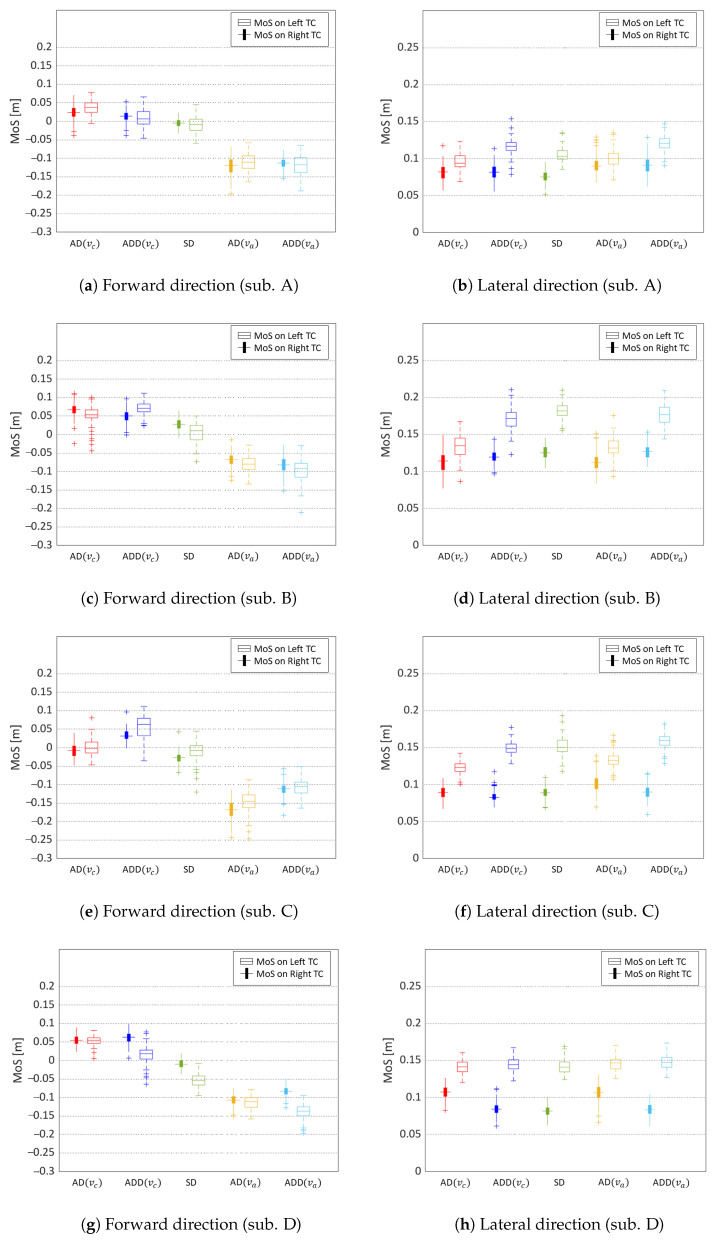

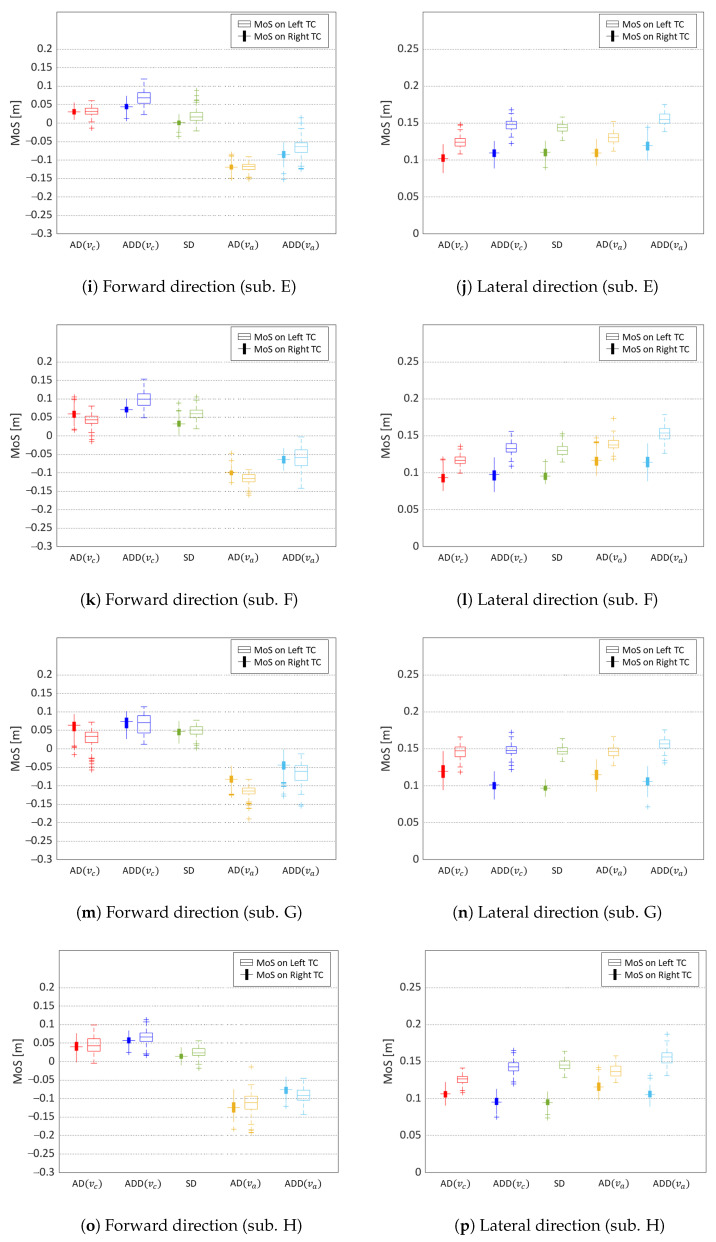

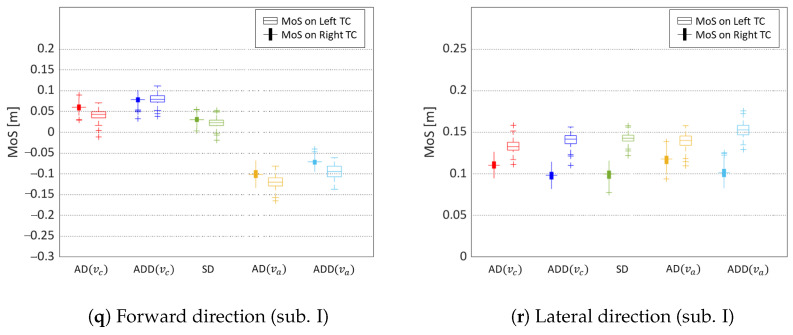
The MoS of each condition. The box plot displays the quartile values, with the cross marker representing outliers.

Figure 2 shows the MoS in the forward and lateral directions for a representative participant. The forward MoS significantly decreased in the faster speed conditions (AD ($v_{a}$) and ADD ($v_{a}$)) for all participants. In contrast, the lateral MoS did not show an effect of speed differences but consistently exhibited asymmetry, with the left step displaying higher values than the right step across all conditions and participants. However, the asymmetry in the lateral direction did not differ between conditions.

### 3.2. MSTS Index

**Figure 3:**
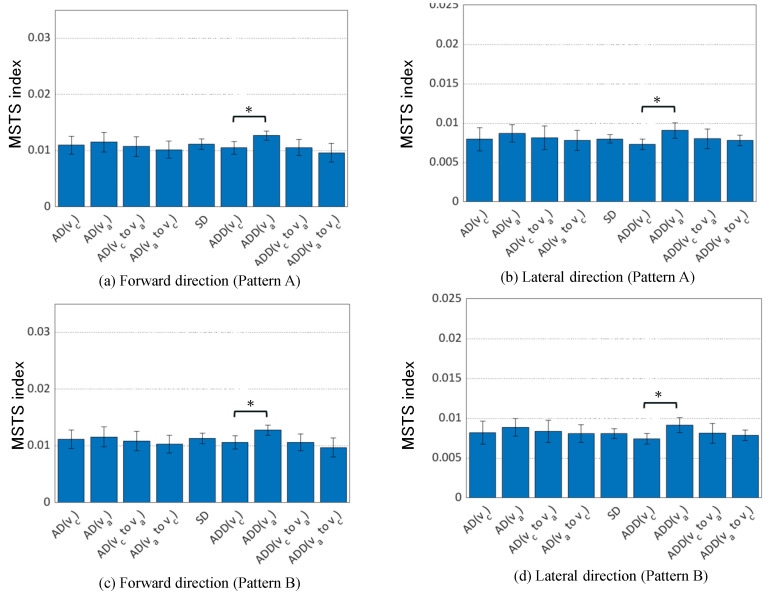
MSTS index comparison across conditions. The bars represent the mean values, with error bars indicating the standard deviation. The asterisk (*) denotes a significance level of 5% (p<0.0071), adjusted using the Bonferroni method.

**Figure 4:**
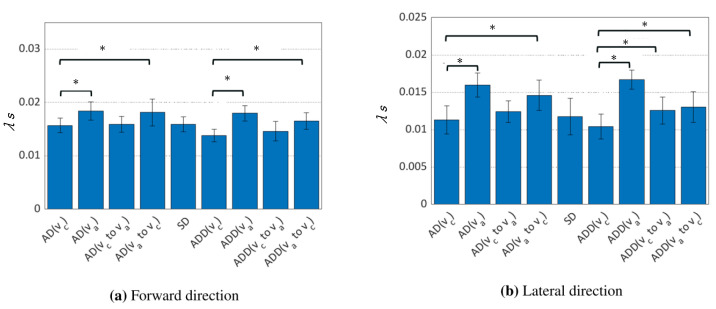
Comparison of the Lyapunov exponent across gait conditions. The bars represent the mean values, with error bars indicating the standard deviation. The asterisk (*) denotes a significance level of 5% (p<0.0071), adjusted using the Bonferroni method.

Figure 3 and Figure 4 show the results of the MSTS index and Lyapunov exponent calculations. The MSTS index, regardless of sensor combination, significantly increased in both the anterior–posterior and mediolateral directions during the ADD condition as steady walking speed increased. The Lyapunov exponent exhibited a similar trend in both the ADD and AD conditions. Additionally, significant differences were observed in conditions involving acceleration and deceleration phases.

**Figure 5:**
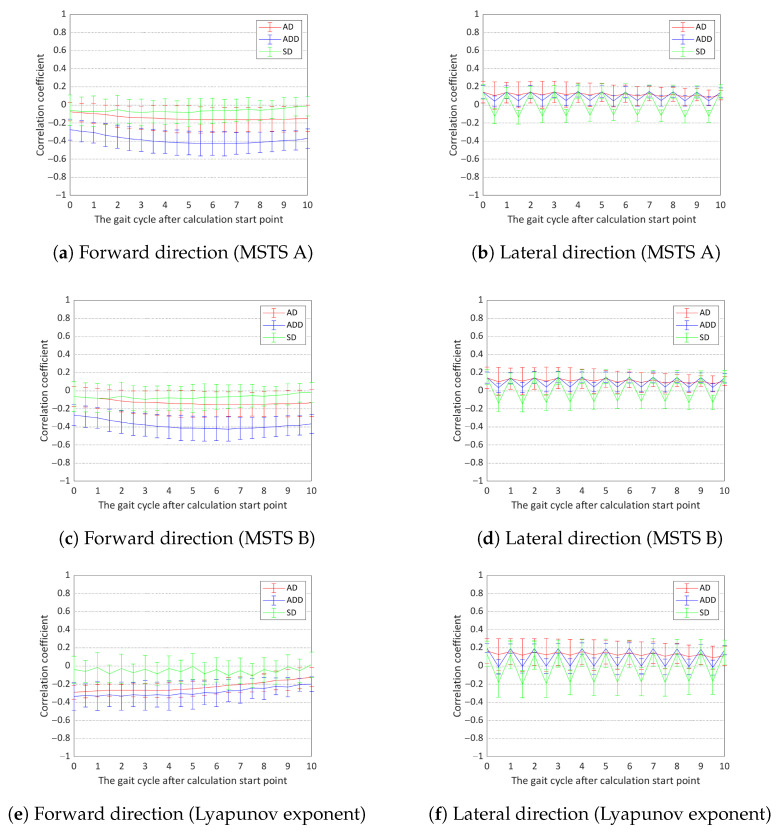
Correlation coefficient between indices and the MoS at the transient phase. The plots represent the mean values, with error bars indicating the standard deviation.

However, as shown in Figure 5, the correlation between the MoS and both the MSTS index and Lyapunov exponent was generally weak. Across the AD ($v_{c}$), ADD ($v_{c}$), and SD conditions, the average correlation values for both directions and indices were below 0.5, with many conditions showing correlations below 0.2.

### 3.3. CNN Classification

In total, 13,261 sets of data were obtained. The results of a binary classification task are detailed, reporting the mean values and standard deviations for both accuracy and recall across different directional thresholds. The classification performance was evaluated using four distinct threshold settings: “Forward (0)”, “Forward (−0.1)”, “Lateral (0.1)”, and “Lateral (0.13)”. For the “Forward (0)” threshold, the model achieved the highest accuracy of 0.948 ± 0.002 and a recall of 0.943 ± 0.004. When the threshold was adjusted to “Forward (−0.1)”, both accuracy and recall decreased to 0.930 ± 0.005 and 0.853 ± 0.038, respectively. In the “Lateral (0.1)” configuration, accuracy and recall were further reduced to 0.919 ± 0.005 and 0.834 ± 0.011, respectively. However, at “Lateral (0.13)”, accuracy improved to 0.933 ± 0.006, while recall returned to 0.943 ± 0.014, matching the recall value obtained at the “Forward (0)” threshold.

The number of samples categorized into “Low” and “High” classes for each directional threshold was counted. For the “Forward (0)” threshold, the number of samples in the “Low” class was 6931, while the “High” class contained 6330 samples, resulting in a relatively balanced distribution. When the threshold was adjusted to “Forward (−0.1)”, there was a notable increase in the “High” class with 10,260 samples, while the “Low” class decreased significantly to 3001 samples. In the “Lateral (0.1)” condition, the sample distribution remained similar, with 3143 samples in the “Low” class and 10,118 in the “High” class. However, for the “Lateral (0.13)” threshold, the distribution shifted again, with a larger proportion in the “Low” class (8116 samples) compared with the “High” class (5145 samples).

## 4. Discussion

### 4.1. Relationship Between Gait Condition and MoS

The comparison of the MoS among speed suggested that an increase in walking speed was associated with a decrease in the forward MoS. This is consistent with the principles of the MoS as a higher walking speed leads to an increase in the velocity of the CoM. In contrast, the lateral MoS showed only minor differences across conditions compared with the forward MoS, but it tended to increase with walking speed. As speed increased, stride time decreased, leading to an increase in cadence. These results align with previous studies [22], which demonstrated a negative correlation between forward MoS and speed and a positive correlation between lateral MoS and cadence. The shorter stride time likely limited the lateral movement of the CoM, contributing to the observed changes in the mediolateral MoS.

In the KAFO-wearing conditions (ADD and SD), reductions in comfortable walking speed and changes in gait patterns were observed. However, there were no clear trends in gait asymmetry for both the forward and lateral MoS. Given the youth and health of the participants, it is possible that they compensated for the restricted knee extension caused by the orthosis, maintaining symmetrical gait patterns. Furthermore, the use of a treadmill, which restricts speed fluctuations, may have helped mitigate asymmetry.

Interestingly, all participants exhibited asymmetry in the mediolateral MoS, regardless of whether they were wearing the orthosis. Although factors such as marker placement, musculoskeletal model bias, and coordinate system misalignment were ruled out, the exact cause of this asymmetry remains unclear.

### 4.2. MSTS Index and Maximum Lyapunov Exponent

The correlation analysis between the MSTS index, Lyapunov exponent, and MoS revealed no strong correlations between these metrics and the MoS. The result suggested that the Lyapunov exponent significantly increased with walking speed (i.e., $v_{a}$ conditions), regardless of orthosis use, while a significant increase in the MSTS index was observed only in the KAFO-wearing conditions. This is the trend that is commonly reported in previous studies [19,20,27]. This suggests that increased walking speed reduces periodic stability. On the other hand, the increase in mediolateral MoS with speed in the KAFO-wearing condition implies that higher speeds may improve kinematic stability. These findings suggest that the MSTS index and Lyapunov exponent, which focus on periodic stability, and the MoS, which evaluates kinematic stability, represent different aspects of gait stability.

The comparison of the MSTS index among conditions suggested that the MSTS index increased significantly in both the anterior–posterior and mediolateral directions with increased walking speed in the orthosis-wearing conditions, regardless of sensor combinations. This suggests that the MSTS index can detect changes in steady walking speed under specific conditions. However, the Lyapunov exponent showed significant differences in more patterns than the MSTS index according to the result. The higher dimensionality used in calculating the MSTS index may have overshadowed information from individual body parts, diminishing its sensitivity to specific body part motions compared with the Lyapunov exponent.

### 4.3. CNN Classification

Although accuracy exceeded 90% in all patterns, variations in threshold values caused significant differences in recall, even for the same direction. The forward (−0.1) and lateral (0.1) thresholds had lower recall due to the smaller proportion of data being classified as “Low”. This data imbalance likely led to insufficient learning for the “Low” category, resulting in lower recall values. Addressing this data imbalance, as well as moving toward non-binary MoS estimation, could improve the quality of gait assessment.

### 4.4. Feasibility of Gait Assessment in Daily Environment

For online gait assessment, multiple IMUs should be attached to lower limb segments to capture and compute gait indices in real time. Our methods indicate that changes in MoS and gait speed can be effectively detected. However, a notable issue remains with the lag in the MSTS index response to speed changes, as evidenced by the indices’ values during transitional phases.

A decreased MoS may be indicative of an increased fall risk, although the relationship between MoS and gait stability requires further investigation. Additionally, environmental variability should be accounted for when assessing fall risk. While IMUs demonstrate robustness against natural environmental factors, such as temperature fluctuations, variations in daily living situations need to be identified. Specifically, accurate gait identification is critical for implementing these gait assessment methods effectively.

### 4.5. Limitations

In this study, neither the MSTS index nor the MoS estimation utilized angular velocity data from the IMUs. Although the MoS calculation only required horizontal velocity components, incorporating angular velocity data could potentially improve accuracy in CNN-based analysis. Additionally, the MSTS index calculation does not impose restrictions on the parameters used, suggesting that adding angular velocity data could enhance the index’s sensitivity.

This study used five or six IMUs for index calculation and MoS prediction. Although IMU measurements are easier to conduct compared with optical motion capture systems, wearing multiple sensors in daily living environments can still be burdensome. Future research should aim to reduce the number of sensors needed for accurate index calculation without compromising performance.

In this study, gait motion was recorded on a treadmill to control gait speed. Given the differences in acceleration waveform patterns between overground and treadmill walking, the proposed method should also be tested in overground conditions. Additionally, variations in gait patterns between treadmill and overground walking should be taken into consideration.

## 5. Conclusions

This study developed a set of indices to evaluate gait stability in daily living environments using IMUs, which impose a low burden on participants. The Lyapunov exponent and MSTS index were calculated from acceleration time-series data, while MoS was selected as the indicator of kinematic stability. The relationship between these indices and the MoS was examined, and a CNN was employed to estimate the magnitude of the MoS directly from the acceleration time-series data.

To validate this approach, treadmill gait experiments were conducted under varying speed conditions and with joint range-of-motion restrictions using an orthosis. Both the Lyapunov exponent and MSTS index captured changes in walking speed, but their correlation with the MoS in both the forward and lateral directions was weak. Given the stabilized nature of treadmill gait, it is recommended that experiments be conducted on an overground walking path. However, the CNN-based estimation achieved over 90% accuracy in predicting MoS in both directions.

This study demonstrated the feasibility of classifying the MoS, a critical indicator of kinematic stability, in both the forward and lateral directions using wearable IMUs. The simplified measurement system makes it suitable for use in daily living environments, offering manufacturers and users of gait support devices a way to quantitatively evaluate device performance beyond basic gait assessment. As more gait parameters can be estimated using IMUs, this achievement is expected to enable a broader range of gait evaluations, including assessments of kinematic stability. Furthermore, the potential of the proposed method to reduce the number of IMUs is significant for the practical implementation of wearable gait assessment devices in societal applications.

## Figures and Tables

**Table 1:** Walking speed of each condition.

	AD ($v_{c}$) [km/h]	ADD ($v_{c}$)	AD ($v_{a}$)	ADD ($v_{a}$)	SD
Subject A	3.5	3.3	5.5	5.3	3.5
Subject B	3.4	3.0	5.4	5.0	3.4
Subject C	3.2	2.5	5.2	4.5	3.2
Subject D	3.0	2.5	5.0	4.5	3.0
Subject E	3.3	2.5	5.3	4.5	3.3
Subject F	3.0	2.5	5.0	4.5	3.0
Subject G	3.0	2.5	5.0	4.5	3.0
Subject H	3.2	2.5	5.2	4.5	3.2
Subject I	3.2	2.6	5.2	4.6	3.2

## Data Availability

The numerical data from the gait experiment are stored by the corresponding author. They will be provided upon request via email.

## Abbreviations

The following abbreviations are used in this manuscript:

IMU	Inertial measurement unit
MoS	Margin of stability
CNN	Convolutional neural network
KAFO	Knee–Ankle–Foot orthosis
AD	Acceleration and deceleration
ADD	Acceleration and deceleration with device
SD	Steady walking with device
CoM	Center of mass
TC	Toe contact
HC	Heel contact
MSTS	Multi-site time-series
